# Applicability of Controllable Normal Force Platform for Study of Bacteria Removal During Dry Cleaning in Dry Food Manufacturing Environments

**DOI:** 10.3390/foods14203459

**Published:** 2025-10-10

**Authors:** Jincheng Ma, Curtis L. Weller, Shaojin Wang, Yu Liu, Zhipeng Liu, Long Chen

**Affiliations:** 1 College of Mechanical and Electronic Engineering, Northwest A&F University, Yangling 712100, China; 2 Department of Biological Systems Engineering, University of Nebraska-Lincoln, Lincoln, NE 68583, USA; cweller1@unl.edu; 3 Department of Food Science and Technology, University of Nebraska-Lincoln, Lincoln, NE 68588, USA; 4 Department of Biological Systems Engineering, Washington State University, Pullman, WA 99164, USA

**Keywords:** dry cleaning, dry food manufacturing environment, cross-contamination, food contact surface, low-moisture foods

## Abstract

Microbial safety in low-moisture foods (LMFs) has attracted widespread public attention due to the multiple outbreaks and recalls in recent years. Dry cleaning methods are typically used in LMFs production environments. However, there is no standardized and consistent method for controlling normal force and measuring the shear force of cleaning tool applied on food contact surfaces during dry cleaning. A dry-cleaning platform with the normal force controllable feature was custom-designed, and its performance was evaluated as the primary objective of the study. Effects of various factors (bacterial type, surface material, surface roughness, and normal force) on the shear force and removal of *Salmonella enterica* Enteritidis PT 30 (*S.* PT 30) and *Enterococcus faecium* NRRL B2354 (*E. faecium*) during dry wiping were investigated using the developed platform. The performance evaluation indicated that the platform was adequately stable during standardized and consistent dry cleaning. Surface roughness, normal force, and surface material significantly affected shear force (*p* < 0.05) applied on food contact surfaces. The bacterial type significantly affected the shear force on stainless steel (*p* < 0.05). No significant difference (*p* > 0.05) was observed in removing *S.* PT 30 from inoculated surfaces after dry wiping under all investigated conditions. Surface material significantly affected the removal of *E. faecium* (*p* < 0.05). The removal of *E. faecium* was numerically higher than that of *Salmonella* under the same conditions. Thus, *E. faecium* may not be a suitable surrogate for *S.* PT 30 removal at the end of dry cleaning under the wiping conditions tested. The potential applications of the platform were also discussed for future studies on how to enhance dry cleaning efficiency. Shear force can guide the disruption of cohesion and adhesion in surface microorganisms/residues, thereby enhancing cleaning efficiency. The custom-designed dry-cleaning platform with the controllable normal force feature has potential applications in further laboratory dry cleaning studies.

## 1. Introduction

Low-moisture foods (LMFs) are historically safe but have recently come under significant scrutiny for their microbial safety, following multiple outbreaks and recalls [1,2,3,4,5,6]. *Salmonella* spp. stands as the major foodborne pathogen in LMFs due to its tolerance to desiccation and ability to remain stable in dry environments [7,8,9,10,11,12]. One of the primary causes of *Salmonella* cross-contamination in LMFs is the inadequate sanitation of equipment surfaces [13,14,15,16].

Wet sanitation is widely used in the food industry and typically introduces water and disinfectants to remove pathogens and allergens from processing environments [17]. However, it is essential to prevent the presence of water in LMFs environments, as introducing water may favor the growth and transfer of pathogens, thereby affecting the safety and quality of LMFs [5,17]. A dry cleaning method is defined as employing physical strategies to reduce the microbial load and remove food residues from food contact surfaces without introducing water [18,19,20]. In the last few decades, studies have been conducted to evaluate the performance of physical dry cleaning methods, although some of these methods are limited to laboratory tests or pilot scale. Dry cleaning methods are defined as employing physical strategies to reduce the microbial load and remove food residues from food contact surfaces without introducing water [18,19,20,21]. He et al. [22] investigated the removal of fruit powder residues from stainless steel by brushing and found that water activity affected the removal effectiveness of the brush. The effects of brushing and scraping on removing wheat flour and non-fat dry milk powder from stainless steel surfaces were investigated by Chen et al. [23], and the effects of food properties, surface roughness, and relative humidity on removal were evaluated. Daeschel et al. [24] used a Monte Carlo simulation-based risk assessment model to evaluate the effects of material flushing and dry wiping on the removal of *Salmonella enterica* Enteritidis PT 30 (*S.* PT 30) from milk powder contact surfaces, and both methods reduced the amounts and concentrations of contaminated milk powder. Liu et al. [20] developed an air impingement dry cleaning device and investigated the effects of relevant factors on the removal of milk powder from stainless steel surfaces, the water activity, residue thickness, nozzle diameter (ND), the ratio of nozzle height from the SS surface to the ND, and air pressure all significantly affect the removal time. Wiping is one of the most common dry cleaning methods [24,25,26].

Magens et al. [27] explained the relationship between the cohesion and adhesion of residues and shear force (horizontal forces) during removal. If cohesion is less than adhesion, during the increase in shear force, the residues would be partially removed at the shear plane (cohesive failure). Conversely, it is completely removed (adhesive failure). However, in practice, both failure modes may occur simultaneously (mixed failure) [20]. Quantifying surface adhesion and cohesion, and shear forces during dry cleaning can maximize the removal of surface residues/microbes and enhance dry cleaning efficiency by increasing shear forces. Research on the influence of various factors on shear force can guide how to effectively enhance shear force during dry cleaning processes, thereby improving cleaning efficiency.

Koo et al. [28] investigated the removal efficiency of *Listeria monocytogenes* from stainless steel plates and Formica^®^ laminate (Cincinnati, OH, USA) surfaces using different cleaning cloths held by hands wearing sterile gloves. They found that the fabric material of the cloths had varied effects on the removal of bacteria and food residues. Additionally, normal and shear forces were not controlled or measured during their manual cleaning process. Mizuno et al. [29] evaluated the effect of manual wiping (dry/wet) on *Bacillus subtilis* endospore removal from stainless steel surfaces. Contact load (normal force) was measured by a pressure sensor during movement of a SS tube wrapped with a 7 cm × 7cm BEMCOT™ cloth (Asahi Kasei Corporation, Tokyo, Japan) to simulate wiping [29]. No correlation was made between contact loads and the force exerted by a human hand under the real removal experimental conditions, and shear force was not measured in the study [29]. In the above studies, the normal and shear forces applied when wiping manually were likely not consistent and standardized. Chen & Snyder [26] used a custom-designed dry-cleaning platform to remove *Salmonella enterica* Enteritidis PT 30 (*S.* PT 30) and potential surrogates from food contact surfaces. While the platform was adjustable in height, it did not specifically measure and control the normal and shear forces applied on food contact surfaces. Prior studies lacked standardized and instrumented control/measurement of normal and shear forces during dry wiping in laboratory research. Normal force and shear force are critical factors for evaluating microbial cross-contamination and dry cleaning efficiency during cleaning [30,31]. Therefore, a platform with a feature to control and measure dynamic normal and shear forces between a cleaning tool and a food contact surface during a dry cleaning process should be developed as a benchmark tool to determine their effects, along with those of other factors (e.g., bacteria type, surface material, and surface roughness), on bacteria removal.

The objectives of this study were (1) to fabricate a dry-cleaning platform for laboratory use with the capability to control normal force, measure shear force and evaluate its performance (stability), and (2) to investigate the effects of relevant factors (surface material, surface roughness and normal force) on the shear force applied by the dry cleaning tool and the removal (i.e., reduction in number) of *S.* PT 30 and its potential surrogate *Enterococcus faecium* NRRL B2354 (*E. faecium*) from food contact surfaces during dry cleaning.

## 2. Materials and Methods

### 2.1. Fabrication of a Normal Force Controllable Dry-Cleaning Platform

#### 2.1.1. Platform Development

**Figure 1:**
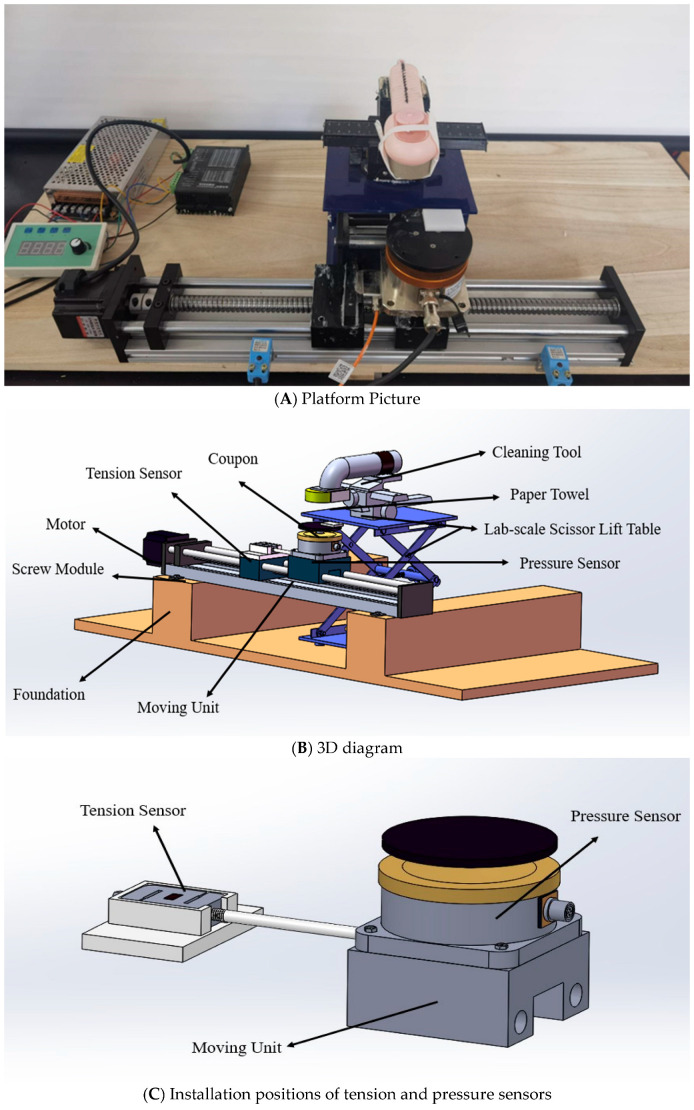
Custom-designed dry-cleaning platform with the normal force controllable feature. (**A**) Platform picture. (**B**) 3D diagram. (**C**) Installation positions of tension and pressure sensors.

The fabricated dry-cleaning platform is shown in Figure 1.

**Figure 2:**
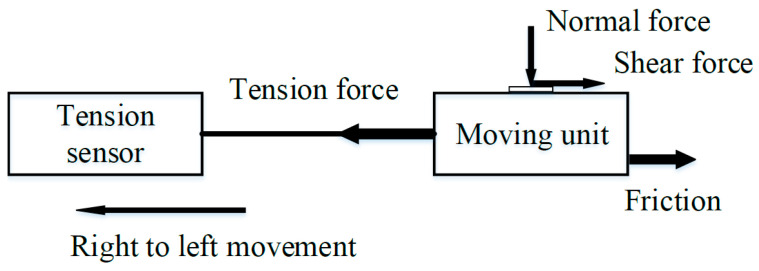
The relationship between shear force, friction, and tension force.

The rig consisted of a screw module including a moving unit (Haijie Jiachuang Technology Co., Beijing, China), a motor and speed controller (Haijie Jiachuang Technology Co., Beijing, China) for the screw, a lab-scale scissor lift table (Bkmamlab Store, Changde, China), a foundation support for the screw module, a pressure sensor, a tension sensor, and sensor data acquisition system for converting the digital signal from the sensor output into force (N) (Pressure Sensor: JLBS-M1; Tension Sensor: JHBS; Sensor data acquisition system: DNXS. Bengbu Sensor System Engineering Co., Ltd., Bengbu, China), and a cleaning tool (a dry water-absorbent sponge brush (length 25 cm × width 5.5 cm; Mrclean Store, Jinhua, China) onto which paper towels (BR060, C&S Paper Co., Ltd., Guangzhou, China) were affixed using removable low-tack tape as shown in Figure 1A). The moving unit was used for reciprocating motion under the action of limit switches (Bengbu Sensor System Engineering Co., Ltd., Bengbu, China). Distance between two limit switches was 300 mm. Paper towels were affixed to the cleaning tool, and the height-adjustable scissor lift table was used to control the position of the cleaning tool to apply selected normal forces and ensure consistent contact between the paper towel and the deposits on a coupon surface (Figure 1B). The sensors were fixed onto the moving unit using epoxy resin adhesive (5800 metal special glue, Shenzhen Chenqi Trading Co., Guangzhou, China) and placed for 24 h to cure after their calibration (Figure 1C). A vertically downward normal force was applied through the paper towel and onto the coupon being cleaned. Shear force between paper towels and inoculum deposits on coupons in the direction opposite to the moving unit travel direction was generated on the deposits during dry cleaning (Figure 2). The sensors were used to measure and transmit observed normal and tension forces during dry cleaning (Figure 2).

#### 2.1.2. Platform Performance Evaluation

The platform performance was evaluated to assess the relationship between friction force and applied normal loads, as well as to determine if any signal noise was introduced during the motion of the screw and cleaning tool before conducting any cleaning experiments. In the experimental dry cleaning process of this study, shear force (*F_S_* in N) during dynamic simulated cleaning was assumed to be the difference between observed tension force (*F_T_* in N) and calculated friction force (*F_f_* in N) between the screw module and the moving unit as described by Equation (1):

(1) $$F_{T}\;=\;F_{S}\;+\;F_{f}$$

However, in the platform performance evaluation, *F_S_* did not exist because only static normal loads were applied to the pressure sensor during the right-to-left movement (Figure 2). The static constant loads (i.e., standard weights of 0, 50, 150, 250, 350, and 450 g) were applied to the pressure sensor while the moving unit moved unidirectionally from right to left at a speed of 19.2 mm/s to assess the system’s performance. Thus, *F_f_* was equal to *F_T_* between the screw module and the moving unit.

Three unidirectional runs of the moving unit were conducted at each of the constant applied static normal loads to record tension sensor values. Then, assuming *F_f_* values were recorded, *F_T_* values, the mean *F_f_* at each constant applied static load was used to fit a linear function for determining the *F_f_* between the screw module and the moving unit under different loads, in which the x-axis is the force of the applied load *F_N_* (N) and the y-axis is *F_f_* (N). The linear model used to estimate *F_f_* for an applied load by the cleaning tool for the 19.2 mm/s speed used during cleaning was given by Equation (2):

(2) $$F_{f}\;=\;0.074\;N\;+\;0.021\;\times \;F_{N}\;$$

Platform stability was also evaluated by recording instantaneous outputs of the tension sensor and pressure sensor over time in two-second intervals for unidirectional travel of the moving unit under loads of 0 N (unloaded) and 4.41 N (maximum normal force steadily applied by the height-adjustable platform) after the motor was turned on (starting from one second, ending at eleven second). The experiment was repeated three times under the same load. The output digital signals were transmitted and collected on a laptop (Xiaoxin Air 14, Lenovo, Shanghai, China) for three runs at each load. The stability of *F_f_* over run time for each load was determined.

### 2.2. Surface Inoculation and Physical Bacterial Removal

#### 2.2.1. Surface Materials

Common LMFs contact surface materials were selected: High-Density Polyethylene (HDPE, Shore hardness of 92 ± 2 HA (provided by manufacturer), Shanghai Boxin Industry Co., Shanghai, China), food-grade Nitrile Rubber (Rubber, Shore hardness of 60 ± 5 HA (provided by manufacturer), Shanghai Lailie Rubber & Plastic Products Co., Shanghai, China), and polished 304 Stainless Steel (SS, Zhongzhiyuan Stainless Steel Products Co., Taizhou, China). The materials were cut into 35 mm L × 24 mm W × 5 mm H coupons. The weights of the SS, HDPE, and Rubber coupons were about 30, 7, and 7 g, respectively.

Since the surface roughness is a relevant variable to consider for food safety [23], and the cleanability is directly influenced by surface roughness [19], the SS coupons were finished with either 80, 400, or 1000 grit sandpapers (Hua Grinding Abrasives Co., Guangzhou, China) to provide the coupons with three surface roughnesses (low, medium, and high). Mean surface roughness (Ra, μm) values for the three types of coupons were determined using a portable profilometer (JITAI-810, Jitai Technology, Huzhou, China) with a trace length of 20 mm for triplicate measurements per coupon per type of roughness. The low surface roughness of coupons finished with the 1000 grit sandpaper was 0.6 ± 0.1 μm (using sandpaper with a higher grit number for finishing would not significantly decrease the surface roughness), lower than 0.8 μm of Ra-value, which is the maximum surface roughness acceptable for hygienic design according to guidelines and standards of organizations such as the European Hygienic Engineering and Design Group [32]. However, in practice, improper use of tools (such as scrapers) may cause abrasions or scratches on SS surfaces, thereby increasing surface roughness. The crevices on the SS food contact surface might eventually serve as a substrate for pathogen harborage and growth. High surface roughness with the 80 grit sandpaper (1.6 ± 0.1 μm) was the maximum roughness achieved in preliminary experiments in this study, representing the most severe abrasion of contact surfaces. The median with the 400 grit sandpaper (1.1 ± 0.1 μm) between the low and high roughness values was selected as the medium surface roughness. Similar surface roughnesses (0.47, 1.07, and 1.34 μm) for *E. faecium* survival and transfer studies have been reported by Lv et al. [33].

The hardness of SS coupons was measured using a Rockwell hardness tester (XHR-150A, Xinhechuang Instrument Co., Ltd., Jinan, China) equipped with a diamond cone indenter with a test force of 60 kgf and recorded in HRA, as the hardness of SS was not suitable for Shore hardness measurement, which was only suitable for soft or elastic materials. The mean hardness of SS was determined from measurements at three locations on each coupon, taken three times, and the experiments were conducted 3 times.

#### 2.2.2. Bacterial Strains

The microbial strains used in this study were *Salmonella enterica* Enteritidis PT 30 and its potential surrogate *Enterococcus faecium* NRRL B-2354 (*E. faecium*). *S.* PT 30 is a pathogen of concern in LMFs [26,34]. *E. faecium* is a widely used surrogate for *Salmonella* spp. in LMFs [35,36,37,38,39].

#### 2.2.3. Preparation of Inoculum

Inoculum preparation was followed based on previous studies [6]. Both bacteria were stored in frozen culture in TSB (Tryptic Soy Broth, Beijing AOBOX, Beijing, China), supplemented with 20% (*v*/*v*) glycerol, at −18 °C. The individual frozen stock was thawed in a biosafety cabinet before a loop (10 μL) of bacterial solution was transferred into 10 mL of TSB broth in a sterilized 20 mL test tube and incubated overnight at 37 °C. The overnight TSB broth test tube was vortexed for 30 s, and a loop (10 μL) of TSB broth was streaked onto a TSA (Tryptic Soybean-Casein Digest Agar Medium, Beijing AOBOX, Beijing, China) plate and incubated at 37 °C for 24 h. The working stock plates were refrigerated at 4 °C for use within 4 weeks. A single isolated colony from the plate was transferred via loop into 10 mL of TSB broth in a test tube, then incubated overnight at 37 °C. The resulting culture (100 μL) was spread evenly on TSA plates with a single-use plastic spreader (Beijing Labgic Technology Co., Ltd., Beijing, China) for each bacterium. The plates were also incubated at 37 °C overnight. Three mL of 0.9% buffered normal saline solution on the TSA agar surface was used to loosen and harvest the bacterial lawn, resulting in culture suspensions. Each bacterial inoculum was collected using a 1000 μL pipette (Thermo Fisher Scientific Instruments Co., Ltd., Suzhou, China) and placed in a 20 mL test tube. Bacterial concentration and log reduction were both recorded in log_10_ CFU (1og). The initial inoculum concentrations were 10.49 ± 0.15 log CFU/mL for *S.* PT 30 and 9.67 ± 0.11 log CFU/mL for *E. faecium*. The initial concentrations of culture suspension were similar to those reported by Xie et al. [38,40].

#### 2.2.4. Inoculation of Surfaces

Before inoculation, all coupons were sanitized in 10% bleach (Lippo Enterprise Group Ltd., Guangzhou, China) for 5 min, then wiped with sterile paper towels, and soaked in 75% ethanol (Shandong Annjet Disinfection Technology Co., Ltd., Jinan, China) for two minutes [26]. Coupons were then wiped again with sterile paper towels and placed on paper towels in a biosafety cabinet (SW-CJ-2FD, Suzhou Purification Equipment Co., Ltd., Suzhou, China) for overnight air drying. The sanitized coupons of three material types were spot inoculated with eighteen spots (10 μL per spot) of the bacterial inoculum without mixing food residues, in an arrangement of 6 × 3 as described by Ma et al. [41], and achieved a cell concentration of ~9.5 log CFU/Coupon for *S.* PT 30, and ~9.0 log CFU/Coupon for *E. faecium*. After inoculation, the coupons were dried overnight in the biosafety cabinet at an environment relative humidity (RH) range of 28–38% and a temperature range of 25 ± 0.1 °C.

#### 2.2.5. Bacteria Removal Experiments

The bacteria removal experiments were conducted using the custom-designed dry-cleaning platform (Figure 1A). Two sets of three-factor experiments were conducted: (1) two bacterial types (*S.* PT 30 and *E. faecium*) × three surface materials (SS of low surface roughness, HDPE, and Rubber) × three normal force levels (1.0, 2.5, and 4.5 N); and (2) two bacterial types (*S.* PT 30 and *E. faecium*) × three surface roughness (SS, Ra = 0.6 ± 0.1, 1.1 ± 0.1, and 1.6 ± 0.1 μm) × three normal force levels (1.0, 2.5, and 4.5 N). Three biological replicates were performed for each of the three-factor experiments. A single-use paper towel was bound to the removable sponge of the cleaning tool (Figure 1A). Paper towels and the removable sponges were previously autoclaved (LMQ.C, Shandong Xinhua Medical Equipment Co., Zibo, China) at 121 °C for 20 min before each experiment. The surfaces of the dry-cleaning platform were sanitized with 75% ethanol (manufacturing information) and wiped with sterilized paper towels.

The coupons, after drying, were fixed on the pressure sensor carrier surface using double-sided tape (Figure 1B). The height-adjustable scissor lift table was adjusted to one of three heights such that normal forces applied on the coupons were approximately 1.0 (low), 2.5 (medium), and 4.5 (high) N. The dry cleaning process began within one second of when the moving unit was set in unidirectional motion, and the attached single-use paper towel wiped all deposits on the coupon surface. The dry cleaning process for a coupon was repeated three times. The actual stroke length over the coupon was ~55 mm when the coupon contacted the paper towel. The mean shear force ${\bar{F}}_{S_{X}}$ for each of the dry cleaning runs was calculated using Equation (3):

(3) $${\bar{F}}_{S_{X}}=\frac{\sum_{i\;=\;1}^{n}\left(F_{T_{i}}\;-\;F_{f_{i}}\right)}{n}$$

where $F_{T_{i}}$ is the detected tension force recorded at the *i*th two-second interval during a dry cleaning run, and $F_{f_{i}}$ is the friction force estimated using Equation (2), in which the mean normal force ${\bar{F}}_{N_{X}}$ for each of the dry cleaning runs was calculated using Equation (4):

(4) $${\bar{F}}_{N_{X}}=\frac{\sum_{i=1}^{n}\left[F_{N_{i}}\;-\;(\frac{m_{coupon}\;\times \;g}{g_{c}})\right]}{n}$$

where $F_{N_{i}}$ is the applied normal load at the *i*th two-second interval during a dry cleaning run, *m_coupon_* is coupon mass (e.g., SS at 0.03 kg, and HDPE or Rubber at 0.007 kg), *g* is gravitational acceleration (9.81 m/s^2^), *n* is the number of runs, and *g_c_* is the conversion factor (1 kg·m/N·s^2^).

The coupons before and after dry cleaning were collected into individual sample bags. Ten mL of 0.9% buffered normal saline solution was added to each sample bag, and the coupons in the sample bag were rubbed by hand for two minutes. After a series of tenfold dilutions, 100 μL samples were withdrawn from each dilution in duplicate and spread onto TSA plates for overnight incubation (LRH-250A, TAIBAO Medical, Guangzhou, China), before counting the colonies. The log reduction in the number of microbes after dry cleaning $Log_{i}$ was calculated based on Equation (5):

(5) $$Log_{i}=Log_{inoc_{i}}-Log_{c\ln d_{i}}$$

where $Log_{inoc_{i}}$ is the log microbial count on the coupon before the *i*th dry cleaning run, and $Log_{c\ln d_{i}}$ is the log microbial count on the coupon after the *i*th dry cleaning run.

### 2.3. Experimental Design and Statistical Analysis

(1) Bacterial type, surface roughness, and normal force, and (2) bacterial type, surface material, and normal force were treated as predictor variables, individually. Shear force and bacterial log reduction were treated as numerical response variables. The shear force and log reduction data were log-transformed, and both conformed to normality and homoscedasticity. However, the original data is retained in Table 1, Table 2, Table 3 and Table 4. Three-way ANOVA was used to determine statistical significance and evaluate the interaction effects among the predictor variables at the 95% confidence interval (α = 0.05) using R Studio (R version 4.4.1, 2024). Two-way ANOVA was also used by dividing the microorganisms into two blocks, considering that there might be inherent differences between the two microorganisms to isolate and remove from the experimental error, thereby increasing the precision of the estimates for the effects of predictor variables across the microorganisms. The results of ANOVA were shown in Tables S1–S12. Significant effect found for any predictor variable resulted in the use of Tukey’s multiple comparison test. The 95% confidence intervals for the significance factors were calculated in R Studio.

## 3. Results and Discussion

### 3.1. Performance of the Developed Dry-Cleaning Platform

**Figure 3:**
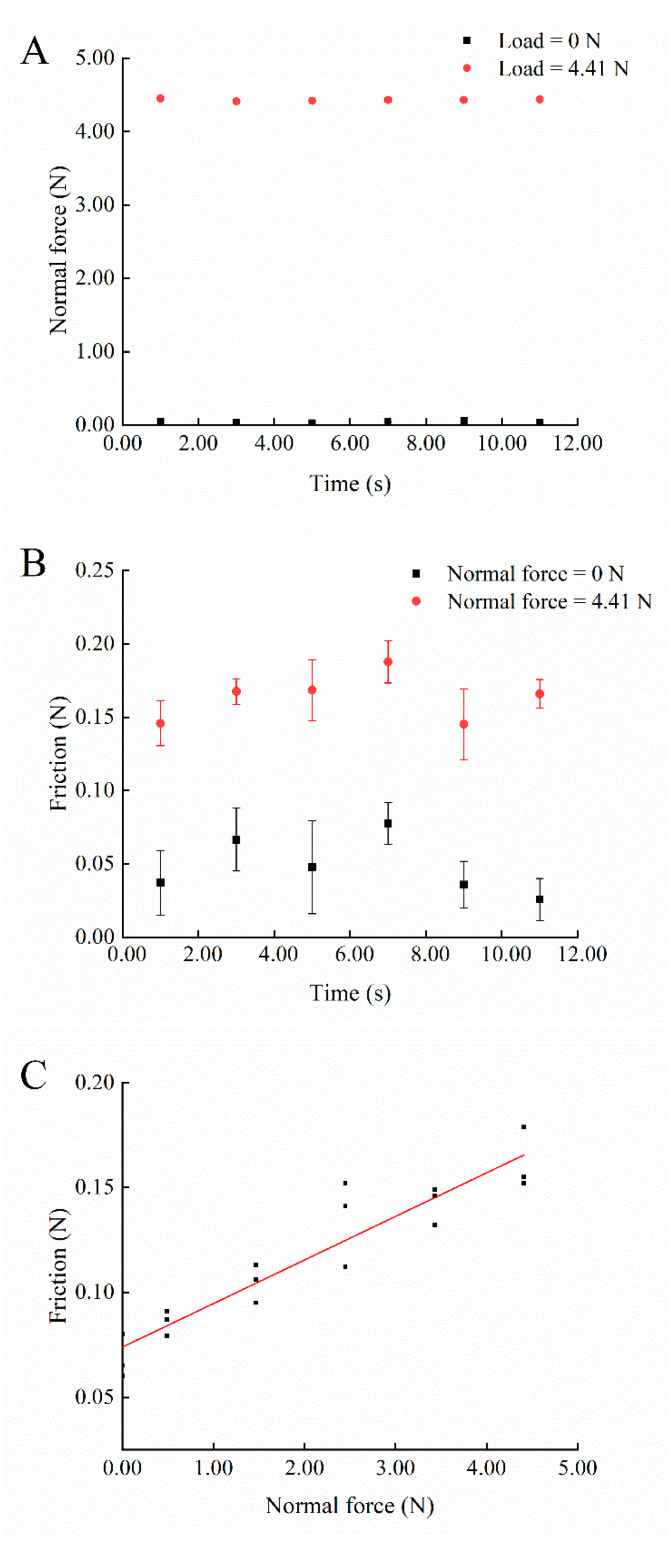
(**A**) Normal force changes with time under loads of 0 N and 4.41 N (the error bar was blocked due to low standard deviations (SD)). (**B**) Friction changes between the moving unit and screw module with time under the load of 0 N and 4.41 N. (**C**) Friction between the moving unit and screw module changes with different normal forces of 0, 0.49, 1.47, 2.45, 3.43, and 4.41 N.

Figure 3A shows the instantaneous normal force. The instantaneous normal force was close to the load value since the measuring plane of the pressure sensor was not affected by external forces except for the constant load. The maximum differences between the normal forces for right-to-left movement and loads across runs were 0.002 N for 0 N and 0.004 N for 4.41 N. This indicated that the pressure sensor could accurately measure the normal force during the dry cleaning process. The measured friction fluctuated up and down when the moving unit moved from right to left, and the changes could be due to the slight oscillation of the moving unit during platform running (Figure 3B). The motor speed might not reach the set speed instantly at startup, which would generate a certain acceleration and affect the stability of the measured friction. But the differences of the maximum and minimum values of the friction from the right to left movement were 0.07 N for the load of 0 N and 0.06 N for the load of 4.41 N, which indicated that the platform was relatively stable. And the friction was very low relative to the shear force during the dry cleaning process. Figure 3C illustrates the mean measured values of the friction during a right-to-left run. The friction was a linear function of normal force (Figure 3C). The moving unit was running at a constant speed (19.2 mm/s) during the test; therefore, the force on the tension sensor was balanced. The measured tension force was equal to the shear force from the surface on which the dynamic normal force was applied and the friction between the screw module and the moving unit when the platform was running (Figure 2). The friction was positively correlated with the normal force; therefore, the friction during platform running was measured when different normal forces were applied.

The R^2^ = 0.880, and RMSE = 0.011 of the fitted model indicated a good linear relationship between normal force and friction during platform running (Equation (2) and Figure 3C). The measured friction increased from 0.07 ± 0.01 to 0.17 ± 0.02 N when the constant normal force increased from 0 to 4.41 N. The friction was much lower compared to the calculated shear force (shown in Table 1 and Table 3). Thus, normal force and shear force were stable, and the normal force was controllable during dry cleaning. The developed platform could stably control the normal force and calculate the shear force for further standardized and consistent dry cleaning experiments.

### 3.2. Bacterial Removal on Three Surface Materials

The shear force and bacterial removal (log reduction) from coupons of three surface materials to single-use paper towels after dry cleaning are summarized in Table 1. And the shear force and bacterial removal means are shown in Table 2.

**Table 1:** Shear force and bacterial removal on SS (Ra, 0.6 ± 0.1 μm), HDPE, and Rubber following dry cleaning with a single-use paper towel.

Microorganism	Surface Material	Normal Force (N)	Count Before Cleaning (log (CFU/Coupon))	Count After Cleaning (log (CFU/Coupon))	Log Reduction (log (CFU/Coupon))	Shear Force (N)
*S.* PT 30	SS	1.10 ± 0.05	9.63 ± 0.09	9.56 ± 0.10	0.06 ± 0.04	0.30 ± 0.02
		2.58 ± 0.03		9.53 ± 0.14	0.09 ± 0.06	0.61 ± 0.01
		4.41 ± 0.10		9.55 ± 0.08	0.07 ± 0.06	0.93 ± 0.01
	HDPE	1.10 ± 0.05	9.58 ± 0.19	9.52 ± 0.27	0.09 ± 0.07	0.13 ± 0.02
		2.58 ± 0.03		9.52 ± 0.25	0.04 ± 0.04	0.27 ± 0.01
		4.41 ± 0.10		9.50 ± 0.22	0.08 ± 0.05	0.43 ± 0.01
	Rubber	1.10 ± 0.05	9.51 ± 0.22	9.50 ± 0.24	0.02 ± 0.05	0.41 ± 0.03
		2.58 ± 0.03		9.53 ± 0.26	0.09 ± 0.05	1.02 ± 0.05
		4.41 ± 0.10		9.50 ± 0.21	0.08 ± 0.05	1.60 ± 0.08
*E. faecium*	SS	1.10 ± 0.05	9.06 ± 0.09	8.97 ± 0.15	0.09 ± 0.07	0.29 ± 0.02
		2.58 ± 0.03		8.92 ± 0.24	0.18 ± 0.09	0.61 ± 0.01
		4.41 ± 0.10		8.88 ± 0.29	0.12 ± 0.13	0.92 ± 0.02
	HDPE	1.10 ± 0.05	9.06 ± 0.11	9.02 ± 0.13	0.03 ± 0.02	0.15 ± 0.01
		2.58 ± 0.03		8.99 ± 0.10	0.09 ± 0.04	0.26 ± 0.02
		4.41 ± 0.10		9.03 ± 0.08	0.07 ± 0.03	0.43 ± 0.03
	Rubber	1.10 ± 0.05	9.05 ± 0.12	9.03 ± 0.03	0.04 ± 0.02	0.41 ± 0.07
		2.58 ± 0.03		8.98 ± 0.03	0.12 ± 0.02	1.04 ± 0.06
		4.41 ± 0.10		9.00 ± 0.05	0.06 ± 0.03	1.56 ± 0.07

Note: Mean ± standard deviation from three replications.

**Table 2:** Shear force and bacterial removal means for SS (Ra, 0.6 ± 0.1 μm), HDPE, and Rubber surfaces during dry cleaning with a single-use paper towel.

Factor	n	Log Reduction Mean (log (CFU/Coupon))	Shear Force Mean (N)
Microorganism Effect			
*S.* PT 30	9	0.08	0.63
*E. faecium*	9	0.09	0.64
Surface Material Effect			
SS	6	0.11	0.61 [0.50, 0.73] ^b^
HDPE	6	0.07	0.28 [0.23, 0.33] ^c^
Rubber	6	0.07	1.01 [0.79, 1.23] ^a^
Normal Force Effect			
Low *F_N_*	6	0.07	0.29 [0.24, 0.34] ^c^
Medium *F_N_*	6	0.09	0.64 [0.49, 0.78] ^b^
High *F_N_*	6	0.10	0.98 [0.76, 1.20] ^a^
Microorganism–Surface Material Interaction Effect			
*S.* PT 30 × SS	3	0.08 [0.04, 0.11] ^ab^	0.62
*E. faecium* × SS	3	0.15 [0.08, 0.22] ^a^	0.61
*S.* PT 30 × HDPE	3	0.09 [0.05, 0.12] ^ab^	0.28
*E. faecium* × HDPE	3	0.05 [0.02, 0.08] ^ab^	0.29
*S.* PT 30 × Rubber	3	0.11 [0.08, 0.13] ^ab^	1.01
*E. faecium* × Rubber	3	0.04 [0.01, 0.08] ^b^	1.00
Surface Material–Normal Force Interaction Effect			
SS × Low *F_N_*	2	0.08	0.31 [0.29, 0.32] ^f^
SS × Medium *F_N_*	2	0.14	0.61 [0.60, 0.62] ^d^
SS × High *F_N_*	2	0.13	0.93 [0.93, 0.93] ^c^
HDPE × Low *F_N_*	2	0.06	0.15 [0.14, 0.16] ^h^
HDPE × Medium *F_N_*	2	0.07	0.27 [0.26, 0.27] ^g^
HDPE × High *F_N_*	2	0.08	0.43 [0.42, 0.44] ^e^
Rubber × Low *F_N_*	2	0.04	0.42 [0.37, 0.47] ^e^
Rubber × Medium *F_N_*	2	0.05	1.03 [0.99, 1.07] ^b^
Rubber × High *F_N_*	2	0.09	1.58 [1.53, 1.63] ^a^
Microorganism–Normal Force Interaction Effect			
*S.* PT 30 × Low *F_N_*	3	0.09	0.29
*E. faecium* × Low *F_N_*	3	0.05	0.29
*S.* PT 30 × Medium *F_N_*	3	0.09	0.63
*E. faecium* × Medium *F_N_*	3	0.08	0.64
*S.* PT 30 × High *F_N_*	3	0.09	0.99
*E. faecium* × High *F_N_*	3	0.11	0.97
Microorganism–Surface Material–Normal Force Interaction Effect			
*S.* PT 30 × SS × Low *F_N_*	1	**/**	**/**
*S.* PT 30 × SS × Medium *F_N_*	1		
*S.* PT 30 × SS × High *F_N_*	1		
*S.* PT 30 × HDPE × Low *F_N_*	1		
*S.* PT 30 × HDPE × Medium *F_N_*	1		
*S.* PT 30 × HDPE × High *F_N_*	1		
*S.* PT 30 × Rubber × Low *F_N_*	1		
*S.* PT 30 × Rubber × Medium *F_N_*	1		
*S.* PT 30 × Rubber × High *F_N_*	1		
*E. faecium* × SS × Low *F_N_*	1		
*E. faecium* × SS × Medium *F_N_*	1		
*E. faecium* × SS × High *F_N_*	1		
*E. faecium* × HDPE × Low *F_N_*	1		
*E. faecium* × HDPE × Medium *F_N_*	1		
*E. faecium* × HDPE × High *F_N_*	1		
*E. faecium* × Rubber × Low *F_N_*	1		
*E. faecium* × Rubber × Medium *F_N_*	1		
*E. faecium* × Rubber × High *F_N_*	1		

Note: Values followed by the same letters within a column for a factor or interaction of factors are not significantly different (*p* > 0.05). **/** means the mean values of log reduction and shear force of three-way interaction can be found in Table 1. n represents the sample size used to calculate the means.

Normal force had a significant effect on the shear force during the dry cleaning process (*p* < 0.05) (Table S1). Shear force significantly increased from 0.29 [0.24, 0.34] N to 0.64 [0.49, 0.78] N to 0.98 [0.76, 1.20] N with increasing mean normal force from low to medium to high, respectively (*p* < 0.05) (Table 2 and Table S1). The shear force was also significantly affected by the surface material (*p* < 0.05) (Table 2 and Table S1). The shear force on Rubber was significantly higher than the shear forces observed for SS and HDPE under the same conditions. Mean shear force for Rubber (1.01 [0.79, 1.23] N) was significantly higher than the mean shear force for SS (0.61 [0.50, 0.73] N), which in turn was significantly higher than the mean shear force for HDPE (0.28 [0.23, 0.33] N) (*p* < 0.05) (Table 2). The highest shear force was 1.60 ± 0.08 N on Rubber, and the shear forces were only 0.93 ± 0.01 N on SS and 0.43 ± 0.03 N on HDPE under the same conditions. The bacterial type inoculated on coupons of three material surfaces had no significant effect on the shear force (*p* > 0.05) (Table S1). There was almost no difference in shear force applied on the coupon surface inoculated with *S.* PT 30 and *E. faecium* under the same conditions. In addition, the interaction between the normal force and the surface material was observed to have a significant effect on the shear force (*p* < 0.05) (Table 2 and Table S1). The highest mean shear force (1.58 [1.53, 1.53] N on Rubber at high normal force) was significantly higher than the mean shear forces on any other materials at any normal forces (Table 2).

Although the microbial log reduction was an important parameter in assessing the efficiency of dry cleaning, normal force, surface material, and bacterial type had no significant effect on removal in this study (*p* > 0.05) (Table S2). It may be due to the high initial inoculation level. Bacterial removals of *E. faecium* were numerically higher than those of *S.* PT 30. For example, the highest log reduction of *E. faecium* on all three materials was 0.18 ± 0.09 log CFU/Coupon; the removal of *S.* PT 30 under the same conditions was 0.09 ± 0.06 log CFU/Coupon. *E. faecium* was also found to be more readily removed from dry food contact surfaces by cleaning tools than *S.* PT 30 [23]. *E. faecium* may not be a suitable surrogate for *S.* PT 30 in bacterial removal during dry cleaning. Higher bacterial removal may overestimate the efficiency of dry cleaning tools. The higher removal may be related to bacterial properties and cell distributions on dry surfaces. Xie et al. [38] investigated the distribution of *Salmonella* cocktail and *E. faecium* on the SS using scanning electron microscopy, also prepared each bacterial inoculum using the lawn-based method, and found that *Salmonella* cocktail and *E. faecium* showed different distributions on the SS surface. The aggregates of *E. faecium* were loosely formed, and the *Salmonella* cocktail formed a compact layer over the underlying texture pattern of SS, which might make it easier for *E. faecium* to attach to the cleaning tool and be detached from the surfaces.

Although the shear force on Rubber was higher than those on SS and HDPE due to material properties, mean bacterial log reductions on SS, HDPE, and Rubber were 0.11, 0.07, and 0.07 log CFU/Coupon (not significant, *p* > 0.05) (Table S2). It may also be due to the high initial inoculation level (>9 log CFU/Coupon). The interaction of bacterial type and surface material significantly affected the bacterial removal (*p* < 0.05) (Table S2). The mean removal of *E. faecium* on SS (0.15 log CFU/Coupon) was significantly higher (*p* < 0.05) than that on Rubber (0.04 log CFU/Coupon) (Table 2). And the ANOVA performed on surfaces inoculated with *E. faecium* showed a significant effect (*p* < 0.05) of surface material on the removal of *E. faecium* (Table 2 and Table S6). The highest *E. faecium* reduction on SS (0.18 ± 0.09 log CFU/Coupon) was higher than those on HDPE (0.09 ± 0.04 log CFU/Coupon) and Rubber (0.12 ± 0.02 log CFU/Coupon). The hardness of three materials was 55.5 ± 1.2 HRA for SS (surface roughness of 0.6 ± 0.1 μm), 92 ± 2 HA for HDPE, and 60 ± 5 HA for Rubber. It had been reported that hard materials were less conducive to bacterial adhesion than soft materials [42]. Although the test methods and units of hardness were not consistent for the three materials in this study, and Shore hardness and Rockwell hardness could not be compared directly, the hardness of SS, if capable of being measured with the Shore HA method, would be approximated to be at the top end of the scale, having a maximum hardness value higher than those of HDPE and Rubber.

### 3.3. Bacterial Removal on SS with Three Surface Roughnesses

The shear force and bacterial log reduction from SS coupons of three surface roughnesses to single-use paper towels after dry cleaning are summarized in Table 3. And the shear force and bacterial removal means are shown in Table 4.

**Table 3:** Shear force and bacterial removal on SS with three different surface roughnesses following dry cleaning with a single-use paper towel.

Microorganism	Surface Roughness (Ra, μm)	Normal Force (N)	Count Before Cleaning (log (CFU/Coupon))	Count After Cleaning (log (CFU/Coupon))	Log Reduction (log (CFU/Coupon))	Shear Force (N)
*S.* PT 30	0.6 ± 0.1	1.10 ± 0.05	9.63 ± 0.09	9.56 ± 0.10	0.06 ± 0.04	0.30 ± 0.02
		2.58 ± 0.03		9.53 ± 0.14	0.09 ± 0.06	0.61 ± 0.01
		4.41 ± 0.10		9.55 ± 0.08	0.07 ± 0.06	0.93 ± 0.01
	1.1 ± 0.1	1.10 ± 0.05	9.48 ± 0.03	9.45 ± 0.09	0.07 ± 0.06	0.33 ± 0.01
		2.58 ± 0.03		9.34 ± 0.11	0.10 ± 0.04	0.64 ± 0.02
		4.41 ± 0.10		9.45 ± 0.12	0.07 ± 0.06	0.95 ± 0.02
	1.6 ± 0.1	1.10 ± 0.05	9.58 ± 0.12	9.58 ± 0.07	0.07 ± 0.03	0.38 ± 0.02
		2.58 ± 0.03		9.55 ± 0.13	0.05 ± 0.03	0.70 ± 0.02
		4.41 ± 0.10		9.50 ± 0.17	0.08 ± 0.05	0.99 ± 0.04
*E. faecium*	0.6 ± 0.1	1.10 ± 0.05	9.06 ± 0.09	8.97 ± 0.15	0.09 ± 0.07	0.29 ± 0.02
		2.58 ± 0.03		8.92 ± 0.24	0.18 ± 0.09	0.61 ± 0.01
		4.41 ± 0.10		8.88 ± 0.29	0.12 ± 0.13	0.92 ± 0.02
	1.1 ± 0.1	1.10 ± 0.05	9.14 ± 0.11	9.08 ± 0.17	0.08 ± 0.05	0.33 ± 0.01
		2.58 ± 0.03		9.10 ± 0.13	0.07 ± 0.04	0.66 ± 0.01
		4.41 ± 0.10		9.11 ± 0.18	0.05 ± 0.05	0.96 ± 0.01
	1.6 ± 0.1	1.10 ± 0.05	8.93 ± 0.19	8.98 ± 0.09	0.12 ± 0.04	0.40 ± 0.01
		2.58 ± 0.03		9.01 ± 0.18	0.06 ± 0.02	0.71 ± 0.01
		4.41 ± 0.10		9.01 ± 0.12	0.06 ± 0.03	1.03 ± 0.05

Note: Mean ± standard deviation from three replications.

**Table 4:** Shear force and bacterial removal means for SS with three different surface roughnesses during dry cleaning with a single-use paper towel.

Factor	n	Log Reduction Mean (log (CFU/Coupon))	Shear Force Mean (N)
Microorganism Effect			
*S.* PT 30	9	0.07	0.65 [0.57, 0.76] ^b^
*E. faecium*	9	0.09	0.66 [0.55, 0.74] ^a^
Surface Roughness Effect			
Low roughness	6	0.11	0.61 [0.50, 0.73] ^c^
Medium roughness	6	0.08	0.65 [0.53, 0.77] ^b^
High roughness	6	0.06	0.71 [0.59, 0.83] ^a^
Normal Force Effect			
Low *F_N_*	6	0.07	0.35 [0.33, 0.37] ^c^
Medium *F_N_*	6	0.09	0.66 [0.64, 0.68] ^b^
High *F_N_*	6	0.08	0.97 [0.95, 0.99] ^a^
Microorganism–Surface Roughness Interaction Effect			
*S.* PT 30 × Low roughness	3	0.08	0.62 [0.45, 0.78] ^d^
*E. faecium* × Low roughness	3	0.15	0.61 [0.45, 0.78] ^d^
*S.* PT 30 × Medium roughness	3	0.10	0.65 [0.48, 0.80] ^c^
*E. faecium* × Medium roughness	3	0.06	0.66 [0.49, 0.82] ^c^
*S.* PT 30 × High roughness	3	0.06	0.69 [0.53, 0.85] ^b^
*E. faecium* × High roughness	3	0.05	0.73 [0.56, 0.90] ^a^
Surface Roughness–Normal Force Interaction Effect			
Low roughness × Low *F_N_*	2	0.08	0.31 [0.29, 0.32] ^h^
Low roughness × Medium *F_N_*	2	0.14	0.61 [0.60, 0.62] ^e^
Low roughness × High *F_N_*	2	0.13	0.93 [0.92, 0.94] ^b^
Medium roughness × Low *F_N_*	2	0.07	0.34 [0.34, 0.35] ^g^
Medium roughness × Medium *F_N_*	2	0.10	0.65 [0.64, 0.66] ^d^
Medium roughness × High *F_N_*	2	0.06	0.96 [0.94, 0.97] ^b^
High roughness × Low *F_N_*	2	0.06	0.40 [0.39, 0.41] ^f^
High roughness × Medium *F_N_*	2	0.04	0.71 [0.70, 0.72] ^c^
High roughness × High *F_N_*	2	0.06	1.02 [1.00, 1.06] ^a^
Microorganism–Normal Force Interaction Effect			
*S.* PT 30 × Low *F_N_*	3	0.07	0.35
*E. faecium* × Low *F_N_*	3	0.07	0.35
*S.* PT 30 × Medium *F_N_*	3	0.09	0.65
*E. faecium* × Medium *F_N_*	3	0.10	0.66
*S.* PT 30 × High *F_N_*	3	0.07	0.96
*E. faecium* × High *F_N_*	3	0.10	0.98
Microorganism–Surface Roughness–Normal Force Interaction Effect			
*S.* PT 30 × Low roughness × Low *F_N_*	1	**/**	**/**
*S.* PT 30 × Low roughness × Medium *F_N_*	1		
*S.* PT 30 × Low roughness × High *F_N_*	1		
*S.* PT 30 × Medium roughness × Low *F_N_*	1		
*S.* PT 30 × Medium roughness × Medium *F_N_*	1		
*S.* PT 30 × Medium roughness × High *F_N_*	1		
*S.* PT 30 × High roughness × Low *F_N_*	1		
*S.* PT 30 × High roughness × Medium *F_N_*	1		
*S.* PT 30 × High roughness × High *F_N_*	1		
*E. faecium* × Low roughness × Low *F_N_*	1		
*E. faecium* × Low roughness × Medium *F_N_*	1		
*E. faecium* × Low roughness × High *F_N_*	1		
*E. faecium* × Medium roughness × Low *F_N_*	1		
*E. faecium* × Medium roughness × Medium *F_N_*	1		
*E. faecium* × Medium roughness × High *F_N_*	1		
*E. faecium* × High roughness × Low *F_N_*	1		
*E. faecium* × High roughness × Medium *F_N_*	1		
*E. faecium* × High roughness × High *F_N_*	1		

Note: Values followed by the same letters within a column for a factor or interaction of factors are not significantly different (*p* > 0.05). **/** means the mean values of log reduction and shear force of three-way interaction can be found in Table 3. n represents the sample size used to calculate the means.

The shear force on SS was significantly affected by the bacterial type (*p* < 0.05) (Table 2 and Table S7). The mean shear force on SS inoculated with *S.* PT 30 (0.65 [0.57, 0.76] N) was significantly lower than that of inoculation with *E. faecium* (0.66 [0.55, 0.74] N) (*p* < 0.05), and the absolute difference in mean shear force for the two bacteria was 0.013 N. The bacterial type may affect the SS surface properties (e.g., surface roughness and friction coefficient), which in turn affect the shear force applied to the SS surface. The surface roughness had a significant effect on the shear force (*p* < 0.05) (Table 4 and Table S7). The rougher the coupon surface, the higher the mean shear force. The shear force increased from 0.61 [0.50, 0.73] to 0.65 [0.53, 0.77] to 0.71 [0.59, 0.83] N) while the surface roughness increased from 0.6 ± 0.1 to 1.1 ± 0.1 to 1.6 ± 0.1 μm (Table 4). Shear force was closely related to the friction between the surface and the paper towel in this study. The friction reduction with decreasing surface roughness has typically been reported for the dry friction regimes and attributed to reduced deformative friction [43]. The interaction of bacterial type and surface roughness significantly affected the shear force on SS (*p* < 0.05) (Table S7). The mean shear force on SS inoculated with *S.* PT 30 (0.73 N) was significantly higher than that of inoculated with *E. faecium* (0.69 N) at high surface roughness (*p* < 0.05) (Table 4). The interaction of bacterial type and surface roughness, surface roughness and normal force also significantly affected the shear force (*p* < 0.05) (Table 4 and Table S7). Mean shear force increased from 0.30 N to 1.03 N on SS (Table 4).

There was no significant difference in the removal of *S.* PT 30 and *E. faecium* on SS surfaces (*p* > 0.05) (Tables S11 and S12). Maximum removal of 0.18 ± 0.09 log CFU/Coupon was observed on the SS of surface roughness (Ra) of 0.6 ± 0.1 μm. The effect of surface roughness on bacterial removal needs to be further explored. It was reported that the cleanability of SS was complicated, which was affected not only by surface roughness but also by surface finish, scratches, and defects [44]. Other factors and interactions not mentioned had no significant effect on either shear force or bacterial removal (*p* > 0.05).

In addition, using the dry spot inoculation method without food residues may underestimate the bacterial removal and efficiency of cleaning tools, because bacteria may attach to surfaces directly and adhere to the surface crevices, making dry cleaning fail to remove a significant amount of bacteria, and food residues might be easier to reach and remove by the cleaning tool. Chen et al. [23] used brushes to remove wheat flour and non-fat dry milk (NFDM) powder from SS and performed allergen testing, and although allergenic residues were still detected after dry cleaning, most residues were removed. Food residues might also change the property of the food contact surface, and change the shear force when cleaning at the same normal force [45]. Bacteria mixed with food residues may also be more easily removed by a cleaning tool. Chen & Snyder [26] used a dry wiping method to remove *S.* PT 30 from SS coupons, and the bacterial log reduction was 0.6 ± 0.4 log CFU/Coupon when using the spot inoculation method, lower than 1.2 ± 0.4 log CFU/Coupon when using the contaminated milk powder inoculation method.

The low reduction may also indicate that a single dry wiping may not be sufficient for dry cleaning purposes. Daeschel et al. [24] used Monte Carlo simulation to model the effects of dry wiping on the removal of *S.* PT 30 and *E. faecium* from SS surface, with an initial level of 4 log, using log reduction as the cells removal of bacteria per wipe, the median number of dry wiping required to remove all *S.* PT 30 on the surface was 9, and *E. faecium* required the number of 4. Even using a moistened cloth, Parvin et al. [25] wiped *Staphylococcus aureus* (ATCC 25923) on a dry polycarbonate coupon with an initial bacterial level of 6.16 ± 0.48 log CFU/Coupon, which was reduced by 1.48 log (96.66%) after 50 wipes, illustrating the difficulty of completely removing bacteria attached to the surfaces.

## 4. Potential Applications of the Developed Dry Cleaning Platform

Although this study was conducted in a laboratory setting, identifying factors affecting surface removal can guide dry cleaning in industrial environments, such as automated robotic arms for dry cleaning, enabling precise control and adjustment of normal force or shear force. The platform may play a key role in standardizing the applications of other dry cleaning processes in the future. The cleaning tool in the platform is interchangeable. If a brush or scraper is fixed on the height-adjustable platform, standardized and consistent dry brushing and scraping cleaning can be performed. Therefore, the platform can be used to study the effects of different types of cleaning tools on removal efficiency. The types of different cleaning tools could provide different normal and shear forces during the dry cleaning process, due to their different contact conditions and material properties. For example, the diameter of bristles, material (nylon, wool, sisal, and steel wire), and stiffness of brushes all could affect the friction and then the shear force on the contact surface. The material of the scraper or cleaning cloth would also have an effect on the shear force under the same normal force during dry cleaning. Tannera et al. [46] investigated the effect of toothbrush stiffness (soft and medium) and brushing force (1, 2, 3, and 4 N) on the cleaning efficacy of bovine dentin samples in vitro using a brushing machine. The cleaning efficacy of the soft-bristle brush was significantly higher than that of the medium-bristle brush at 1 N. And the cleaning efficacy increased with increasing brushing force for both stiffness toothbrushes.

The choice of cleaning tools should be based on specific contamination scenarios, such as the nature of the contaminated surface (surface roughness and friction coefficient, etc.), the physicochemical properties of the food residues, and the specific environmental conditions (temperature and relative humidity (RH), etc.). The physicochemical properties of food residues or environmental conditions could affect the adhesion and cohesion of food residues on surfaces. The normal and shear forces required to remove these food residues vary significantly under different conditions. For example, one application of the platform is to select scrapers based on the type of surface and food residues that need to be cleaned [19]. Middleton et al. [47] suggested that the SS scrapers could be used for rough and raised surfaces, while the plastic scrapers were more suitable for plastic surfaces. Chen et al. [23] found that more brushing passes were needed to clean the NFDM residues on SS surfaces at 0.81 a_w_ than those at 0.16, 0.22, and 0.69 a_w_. And more brushing passes were required to clean NFDM at 0.81 a_w_ than that of wheat flour at 0.80 a_w_. The effect of different types and materials of cleaning tools on cleaning efficiency, the normal force and shear force required to remove food residues with different physicochemical properties on different surface materials, during the dry cleaning processes, could be investigated using the developed platform in the future. Understanding the effects of various factors on the removal of surface microorganisms and residues may aid in the future development of automated cleaning systems in industrial environments, such as automated cleaning robotic arms.

A single dry cleaning method may not be sufficient to remove all surface microorganisms or food residues. The platform can also be used for investigating the cleaning efficiency of a combination of dry cleaning/sanitation methods in LMFs processing environments. Dry sanitation is defined as sanitizing operations applied without using water to reduce microorganisms from surfaces through non-contact physical methods [17]. The dry sanitation methods, for example, dry heat, pulsed light, and ultraviolet light, may be more adept at inactivating surface microorganisms [17,48]. *S.* PT 30 on SS and polypropylene surfaces was reduced by 1.6–4.3 log CFU/cm^2^ with dry heat for 0.5 to 4 h at 90 °C; however, the heat increased the adhesion of food residues on the surfaces, which provided conditions for microbial growth [17]. Dry cleaning methods may be more suitable for removing food residues from surfaces, whether by physical contact or not. Chen & Snyder [26] used dry wiping to remove the residual *Listeria innocua* ATCC 51742 contaminated NFDM from SS surfaces and found the bacterial removal was 1.6 ± 0.3 log. If a pulsed light process is applied after dry wiping, there may be lower chances of bacterial cross-contamination. Chen et al. [23] investigated the effects of brushing and scraping on the removal of NFDM from SS surfaces, considering the physicochemical properties of powdered foods, surface roughness, and the ambient RH. When a_w_ < 0.81, two to four times of brushing or scraping could achieve the cleaning purpose (the mass difference between two consecutive passes of the cleaning tool was <0.5 mg). However, more passes of brushing were required than scraping at 0.81 a_w_ due to NFDM caking. Scraping and brushing can be used in combination to improve the cleaning efficiency in removing high a_w_ food residues like milk powder, which may be superior to the use of a single cleaning tool [23]. The scraper could first be used to remove the strongly adhesive food film for the greater shear force because of its rigid structure, then brushing could remove the remaining particulate on the near surface.

## 5. Limitations

Some limitations were not considered in this study. First, the inoculum levels used were high. High inoculation levels may not accurately reflect real contamination events. A lower inoculation level would help show whether the platform could dry clean effectively. Second, no manual wiping experiments were conducted to benchmark and compare the performance of the developed equipment. Third, using removal as the only criterion to determine whether *E. faecium* could serve as a surrogate for *S.* PT 30 may be insufficient. More microscopic studies, such as bacterial adhesion or distribution, could strengthen this conclusion. Finally, residue testing was not performed, which may render the microbiological experiments unrepresentative of actual dry cleaning conditions. Future studies would consider and address the above-mentioned limitations.

## 6. Conclusions

A dry-cleaning platform that controls normal force to investigate bacterial removal in dry food manufacturing environments was fabricated. The platform was adequately stable to control normal force during a standardized and consistent dry cleaning process. Surface roughness, normal force, and surface material had significant effects on shear force (*p* < 0.05). Bacterial type significantly affected the shear force on SS (*p* < 0.05). The interaction of normal force and surface material, the interaction of bacterial type and surface roughness, and the interaction of bacterial type and surface roughness also significantly affected the shear force applied by the cleaning tool (*p* < 0.05). There was no significant difference in *S.* PT 30 removal at multifactorial and multilevel conditions on all three surface materials during the dry cleaning process (*p* > 0.05). Surface material significantly influenced the removal of *E. faecium* (*p* < 0.05). *E. faecium* may not be a suitable surrogate for *S.* PT 30 removal at the end of dry cleaning under the wiping conditions tested. Investigating shear forces in concert with microbial/residue adhesion and cohesion can enhance surface removal and better guide dry cleaning of low-moisture surfaces. The custom-designed dry-cleaning platform with the normal force controllable feature could provide other potential applications and help further investigations in the dry cleaning area.

## Data Availability

The raw data supporting the conclusions of this article will be made available by the authors on request.

## Supplementary Materials

The following supporting information can be downloaded at: https://www.mdpi.com/article/10.3390/foods14203459/s1, Table S1: Analysis of variance (ANOVA) for log shear force on surfaces of three materials (Three-way interaction); Table S2: ANOVA for log bacterial removal on surfaces of three materials (Three-way interaction); Table S3: ANOVA for log shear force on surfaces of three materials inoculated with *Salmonella* (Two-way interaction); Table S4: ANOVA for log shear force on surfaces of three materials inoculated with *E. faecium* (Two-way interaction); Table S5: ANOVA for log bacterial removal on surfaces of three materials inoculated with *Salmonella* (Two-way interaction); Table S6: ANOVA for log bacterial removal on surfaces of three materials inoculated with *E. faecium* (Two-way interaction); Table S7: ANOVA for log shear force on SS surface with three different surface roughnesses (Three-way interaction); Table S8: ANOVA for log bacterial removal on SS surface with three different surface roughnesses (Three-way interaction); Table S9: ANOVA for log shear force on SS surface with three different surface roughnesses inoculated with *Salmonella* (Two-way interaction); Table S10: ANOVA for log shear force on SS surface with three different surface roughnesses inoculated with *E. faecium* (Two-way interaction); Table S11: ANOVA for log bacterial removal on SS surface with three different surface roughnesses inoculated with *Salmonella* (Two-way interaction); Table S12: ANOVA for log bacterial removal on SS surface with three different surface roughnesses inoculated with *E. faecium* (Two-way interaction).
