# Supplementary material for: Applicability of Controllable Normal Force Platform for Study of Bacteria Removal During Dry Cleaning in Dry Food Manufacturing Environments
Source: Foods. 2025 Oct 10;14(20):3459. doi: 10.3390/foods14203459 (PMC12564543; doi:10.3390/foods14203459)
Supplement: Supplementary file 1 [file foods-14-03459-s001.zip › foods-3891105-supplementary.pdf]

Table S1 Analysis of variance (ANOVA) for log shear force on surfaces of three materials (Three-way interaction)

Factors	Df	Sum Sq	Mean Sq	F-value	Pr (>F)
Normal force	2	2.356	1.178	2033.902	< 0.001
Surface material	2	2.609	1.304	2252.204	< 0.001
Microorganism	1	0.001	0.001	0.218	0.643
Normal force × Surface material	4	0.039	0.010	16.631	< 0.001
Normal force × Microorganism	2	0.001	0.001	0.502	0.609
Surface material × Microorganism	2	0.001	0.001	1.280	0.290
Normal force × Surface material × Microorganism	4	0.004	0.001	1.652	0.183
Residuals	36	0.021	0.001		

Table S2 ANOVA for log bacterial removal on surfaces of three materials (Three-way interaction)

Factors	Df	Sum Sq	Mean Sq	F-value	Pr (>F)
Normal force	2	0.141	0.070	0.343	0.711
Surface material	2	0.304	0.152	0.740	0.483
Microorganism	1	0.371	0.371	1.807	0.187
Normal force × Surface material	4	0.887	0.221	1.079	0.381
Normal force × Microorganism	2	0.324	0.162	0.788	0.462
Surface material × Microorganism	2	1.823	0.911	4.437	0.018
Normal force × Surface material × Microorganism	4	0.060	0.015	0.073	0.989
Residuals	36	7.396	0.205		

Table S3 ANOVA for log shear force on surfaces of three materials inoculated with *Salmonella* (Two-way interaction)

Factors	Df	Sum Sq	Mean Sq	F-value	Pr (>F)
Normal force	2	1.214	0.607	898.130	< 0.001
Surface material	2	1.357	0.678	1003.986	< 0.001
Normal force × Surface material	4	0.014	0.003	5.258	0.005
Residuals	18	0.012	0.001		

Table S4 ANOVA for log shear force on surfaces of three materials inoculated with *E. faecium* (Two-way interaction)

Factors	Df	Sum Sq	Mean Sq	F-value	Pr (>F)
Normal force	2	1.142	0.571	1184.159	< 0.001
Surface material	2	1.252	0.626	1298.863	< 0.001
Normal force × Surface material	4	0.028	0.007	14.587	< 0.001
Residuals	18	0.008	0.001		

Table S5 ANOVA for log bacterial removal on surfaces of three materials inoculated with *Salmonella* (Two-way interaction)

Factors	Df	Sum Sq	Mean Sq	F-value	Pr (>F)
Normal force	2	0.027	0.013	0.069	0.933
Surface material	2	0.343	0.171	0.863	0.438
Normal force × Surface material	4	0.272	0.068	0.342	0.845
Residuals	18	3.581	0.198		

Table S6 ANOVA for log bacterial removal on surfaces of three materials inoculated with *E. faecium* (Two-way interaction)

Factors	Df	Sum Sq	Mean Sq	F-value	Pr (>F)
Normal force	2	0.437	0.218	1.032	0.376
Surface material	2	1.784	0.892	4.209	0.031
Normal force × Surface material	4	0.674	0.168	0.795	0.543
Residuals	18	3.814	0.211		

Table S7 ANOVA for log shear force on SS surface with three different surface roughnesses (Three-way interaction)

Factors	Df	Sum Sq	Mean Sq	F-value	Pr (>F)
Surface roughness	2	0.051	0.025	243.301	< 0.001
Normal force	2	1.829	0.914	8594.942	< 0.001
Microorganism	1	0.001	0.001	6.064	0.018
Normal force × Surface roughness	4	0.008	0.002	19.853	< 0.001
Surface roughness × Microorganism	2	0.001	0.001	8.903	< 0.001
Surface roughness × Microorganism	2	0.001	0.001	0.700	0.503
Normal force × Surface roughness × Microorganism	4	0.001	0.001	2.169	0.092
Residuals	36	0.003	0.001		

Table S8 ANOVA for log bacterial removal on SS surface with three different surface roughnesses (Three-way interaction)

Factors	Df	Sum Sq	Mean Sq	F-value	Pr (>F)
Surface roughness	2	1.008	0.504	1.143	0.330
Normal force	2	0.281	0.140	0.318	0.729
Microorganism	1	0.210	0.210	0.477	0.493
Normal force × Surface roughness	4	2.336	0.584	1.323	0.279
Surface roughness × Microorganism	2	0.817	0.408	0.926	0.405
Surface roughness × Microorganism	2	0.248	0.124	0.281	0.756
Normal force × Surface roughness × Microorganism	4	0.892	0.223	0.505	0.731
Residuals	36	15.880	0.441		

Table S9 ANOVA for log shear force on SS surface with three different surface roughnesses inoculated with *Salmonella* (Two-way interaction)

Factors	Df	Sum Sq	Mean Sq	F-value	Pr (>F)
Surface roughness	2	0.016	0.008	55.998	< 0.001
Normal force	2	0.898	0.449	2970.851	< 0.001
Surface roughness × Normal force	4	0.003	0.001	5.971	0.003
Residuals	18	0.002	0.001		

Table S10 ANOVA for log shear force on SS surface with three different surface roughnesses inoculated with *E. faecium* (Two-way interaction)

Factors	Df	Sum Sq	Mean Sq	F-value	Pr (>F)
Surface roughness	2	0.036	0.018	298.057	< 0.001
Normal force	2	0.931	0.465	7552.677	< 0.001
Surface roughness × Normal force	4	0.005	0.001	23.374	< 0.001
Residuals	18	0.001	0.001		

Table S11 ANOVA for log bacterial removal on SS surface with three different surface roughnesses inoculated with *Salmonella* (Two-way interaction)

Factors	Df	Sum Sq	Mean Sq	F-value	Pr (>F)
Surface roughness	2	0.341	0.170	0.349	0.709
Normal force	2	0.128	0.064	0.130	0.878
Surface roughness × Normal force	4	2.994	0.748	1.530	0.235
Residuals	18	8.804	0.489		

Table S12 ANOVA for log bacterial removal on SS surface with three different surface roughnesses inoculated with *E. faecium* (Two-way interaction)

Factors	Df	Sum Sq	Mean Sq	F-value	Pr (>F)
Surface roughness	2	1.484	0.742	1.887	0.180
Normal force	2	0.401	0.200	0.510	0.608
Surface roughness × Normal force	4	0.234	0.058	0.149	0.961
Residuals	18	7.076	0.393		
